# Measurement of work-related psychological injury with depressive symptoms

**DOI:** 10.1186/s12888-023-05178-w

**Published:** 2023-09-19

**Authors:** Mari Tanaka, Yoshiyuki Hirano, Rieko Takanashi, Noriko Numata, Chihiro Sutoh, Tomohiro Yoshikawa, Eiji Shimizu

**Affiliations:** 1 Department of Cognitive Behavioral Physiology, Graduate School of Medicine, Chiba University, Chiba, Japan; 2 Research Center for Child Mental Development, Chiba University, Chiba, Japan; 3 United Graduate School of Child Development, Osaka University, Kanazawa University, Hamamatsu University School of Medicine, Chiba University and University of Fukui, Suita, Japan; 4 ANA Narita Airport Services Co., Ltd., Narita, Japan; 5 Taimei Corporation Co., Ltd, Kawaguchi, Japan

**Keywords:** Depression, Adjustment disorder, Psychological injury, Work-related stress

## Abstract

**Objective:**

This study aimed to measure the level of psychological injury caused by work-related stress as well as the severity of depression among workers.

**Method:**

First, we conducted an online survey and recruited 500 workers diagnosed with depression or adjustment disorder to investigate what type of stress they experienced within six months before onset. Second, we conducted another online survey and recruited 767 participants who experienced some form of work-related stress. All the participants were classified into four groups by whether or not they were diagnosed with depression and whether or not they quit their jobs due to work-related stress. We used the Impact of Event Scale-Revised (IES-R) to measure psychological injury caused by work-related stressful events and the Patient Health Questionnaire (PHQ)-9 to assess the severity of depression.

**Results:**

In study 1, 62.4% of workers diagnosed with depression or adjustment disorder experienced work-related stress within six months before onset. In study 2, the IES-R mean scores were 40.7 (SD = 23.1) for Group A (workers with depression and quit their jobs) and 36.67 (SD = 23.4) for Group B (workers with depression but stayed at their jobs), with both exceeding the cut-off point (24/25) of PTSD (Post-Traumatic Stress Disorder), while the mean score of Group C (workers who did not have depression but quit their jobs because of work-related stress) was 20.74 (SD = 21.2), and it was 13.89 (SD = 17.4) for Group D (workers who had work-related stress but stayed at their jobs), with both of them below the cut-off point of PTSD. The total scores of IES-R of Group A and Group B were significantly higher than those of Group C and Group D(*p* < 0.001). There was a significant positive correlation between the scores of IES-R and PHQ-9 for all four groups (*r* = 0.708).

**Conclusions:**

This study suggests that it is necessary to measure not only depressive symptoms but also the level of psychological injury resulting from stressful events in the workplace to assess workers with depression.

## Introduction

Depression is a leading cause of disability worldwide and has a significant impact on productivity in the workplace [1], and occupational stress is of increasing importance due to continuing structural changes in the workplace, with both increasing demands and job insecurity imposed on employees [2]. Also, employees who report a lack of decision latitude, job strain, and bullying will experience depressive symptoms over time, and these conditions are amenable to organizational interventions [3]. In Addition, strongly affected by a hard and long working culture known as "workaholic," nearly 200 people are working to death every year in Japan, according to the annual report by the Japanese Ministry of Health, Labor and Welfare, and the financial loss by depression was estimated at approximately $11 billion in 2008 [4]. In 2020, “Act on Comprehensively Advancing Labor Measures, and Stabilizing the Employment of Workers, and Enriching Workers' Vocational Lives” commonly known as “Power Harassment Prevention Law” has been enforced in Japan and all employers are compulsorily required to take measures to protect workers from hazardous working conditions including bullying or harassments at work and psychiatrists and occupational physicians play an important role to support workers with conflicts.

"Psychological injury" is defined as employees' explicit manifestation of distress in the form of affective behavioral and cognitive dysfunction in the work context [5]. Early identification and accurate assessment of psychological injury is an inevitable step in the prevention of serious mental outcomes and it brings benefits not only for employees but also for employers. In Canada, the Mental Health Commission of Canada (MHCC) https://mentalhealthcommission.ca/at-work/ [6] reported that a half-million workers are not able to continue their work due to mental health problems or illness every week, and they publicized a guideline and advised employers to compensate employees' mental injuries. Similarly, the Australian government launched the "Safe Work Australia" website https://www.safeworkaustralia.gov.au/ [7] to advocate the creation of safe working environments and protecting workers' mental health by taking care of work-related mental health conditions. To specifically identify and measure the level of psychological injury, Winwood [8] developed the Psychological Injury Risk Indicator (PIRI), and it has been verified in Italy [9] but not yet in Japan.

It is reported that work-related stress is an independent risk factor for the development of major depressive episodes in the working population [10]. In addition to genetic factors, recent stressful life events have been found to be substantial risk factors for major depression [11]. It has been confirmed that patients with treatment-refractory depression perceive their experiences of onset-related events as severe psychological distress symptoms [12]. Inspired by this finding, we developed the hypothesis that workers with depression had an experience of onset-related psychological injury caused by work-related stress.

Along with this hypothesis, we undertook the following two studies. In Study 1, we investigated what kind of stress workers diagnosed with depression or adjustment disorder experienced within six months prior to onset. In Study 2, we investigated whether workers who developed depression due to work-related stress experienced psychological injury compared to those who had work-related stress but no depression. Additionally, we hypothesized that workers who experienced work-related stress could have difficulties in trusting others and themselves and also in promoting their own mental well-being which means ability to take care of their own mental health.

## Methods

### Study design

Both study 1 and study 2 adopted a cross-sectional design and were approved by the ethics committee of Chiba University Graduate School of Medicine. All the surveys were anonymous and conducted online. We fully explained the purpose and procedure of this study to all participants on the web, and they confirmed their consent to participate in the survey by clicking "I agree" and completing the survey voluntarily.

### Study 1 method

#### Participants and procedure

We recruited 500 participants (250 male, 250 female) who were employed and diagnosed with depression or adjustment disorder under treatment. There was no limit regarding age, occupation and employment style (full time/part time) or duration.

#### Measures

We developed an original questionnaire asking whether or not they experienced private life stress such as divorce, death of family members, or financial crisis and whether or not they experienced work-related stress such as excessive long working hours, workplace bullying, or unexpected accidents at work within six months prior to the onset of depression or adjustment disorder. We also asked if the stressful situation had improved or not by then.

### Study 2 method

#### Participants and procedure

We aimed to collect 200 workers who had work-related depression and quit their jobs (Group A), 200 workers who had work-related depression but stayed on their jobs (Group B), 200 workers who did not have work-related depression but quit their jobs because of work-related stress (Group C), and 200 workers who did not have work-related depression but stayed on their jobs with work-related stress (Group D), totaling 800 participants. There was no limit regarding age, occupation and employment style (full time/part time) or duration. Workers who answered "I haven't experienced any work-related stress" were excluded from this study. We collected demographic data such as age, gender, and occupation from all participants, and psychiatric comorbidity, disease and treatment duration, and age of onset of depression from Groups A and B.

#### Categories of work-related stressful events

The following were the seven categories that we identified as work-related stressful events based on the "Evaluation chart of work-related psychological distress" issued by the Japanese Ministry of Health, Labor and Welfare.

1. Unwanted, forced transfer: It took an enormous amount of time and effort to get used to my new job, as it was an entirely different type of work from the previous one.
2. One-person operation: I was the only person in charge of the operation, and I was constantly overworked with no break or holiday due to the increasing workload.
3. Workplace bullying or assault: I was bullied or harassed or assaulted at work verbally or physically, or psychologically by my co-worker(s). For example, a group of people relentlessly attacked me verbally to degrade and demean my existence.
4. Power harassment: My boss repeatedly harassed me verbally to degrade and demean my existence, and that conduct could not be regarded as supervision.
5. Serious trouble with the boss: I had trouble with my boss regarding management policy, and it affected my daily job performance.
6. Troubles with a colleague or subordinate: I had serious trouble(s) with my colleague or subordinate regarding management policy, and it affected my daily job performance.
7. I had work-related stress other than the above.

#### Measures

##### Impact of event scale-revised

The Impact of Event Scale-Revised (IES-R) is a short, easy self-report measure for assessing the severity of subjective responses to traumatic life events in the past seven days. There are 22 items and 3 subcategories, including 8 items for intrusion, 8 items for avoidance, and 6 items for hyperarousal. Each of the items is scored on a five-point scale (0 = not at all, 1 = a little, 2 = moderately, 3 = a lot, 4 = enormously). The total sum of points ranges from 0 to 88, with higher scores indicating severe post-traumatic stress. The internal consistency and concurrent validity of IES-R were confirmed [13], and the Japanese version of IES-R has been developed and validated [14]. IES-R has been used for screening but not for diagnosis of Post-Traumatic Stress Disorder [15].

We used IES-R to assess the level of psychological injuries caused by work-related stressful events because, as we mentioned in Introduction, there is no scale in our country to assess it and we also intended to see how traumatic their work-related stressful events such as one-person operation or bullying at work were.

All participants were instructed to choose stressful events they experienced at work from the 7 categories we described above, and multiple choices were allowed. Then, they were asked to choose one most stressful work-related event as the targeted traumatic event and answer each item of IES-R specifically about that one. We also asked for their age when it occurred.

##### Patient Health Questionnaire (PHQ)-9

We assessed the severity of depression using the Japanese version of the Patient Health Questionnaire (PHQ-9) [16]. Not only those who were diagnosed with depression but also those without depression were instructed to complete this measure in this study. PHQ-9 is a brief and easy self-administered questionnaire for screening depression, which scores each of the nine criteria as "0" (not at all) to "3" (nearly every day). The PHQ-9 score can range from 0 to 27 since each of the nine items can be scored from 0 (not at all) to 3 (nearly every day). In addition to those nine items, one item was added that asked the participants "how difficult have these problems made it for you to do your work, take care of things at home, or get along with other people?" to assess how difficult their life was due to depressive symptoms.

##### General trust scale [17]

This scale is a 6-item questionnaire that uses general statements to measure participants' beliefs about the honesty and trustworthiness of others in general. This scale adapts a seven-point Likert scale from 1 (completely disagree) to 7 (completely agree). The total points are 42, and a high score means a high tendency of one’s trust in others. All the items are shown as follows.

1. Most people are basically honest.
2. Most people are trustworthy.
3. Most people are basically good and kind.
4. Most people are trustful of others.
5. I am trustful.
6. Most people will respond in kind when they are trusted by others.

##### Mental well-being scale [18]

This 10-item scale is to measure participants' willingness to take care of their own mental health.

This scale adapts a five-point Likert scale ranging from 0 (strongly disagree) to 4 (agree). The total points are 40, and a high score means high motivation for taking care of one's mental health. All the items are shown as follows.

1. I can step back and see the bigger picture.
2. I can control negative feelings such as anger, anxiety, or sorrow.
3. When I think of a negative idea, I can find an alternative one.
4. I can cope with my stress.
5. I think I have cognitive flexibility.
6. I think I have good traits.
7. I can figure out a solution for my immediate problem.
8. My goals are small, but I live my life positively.
9. I have someone whom I'm grateful for.
10. I can find happiness in small things.

### Statistical analysis

We analyzed the data separately for the four groups, workers who had depression and quit their jobs (Group A), workers who had depression but stayed on their jobs (Group B), workers who did not have depression but quit their jobs because of work-related stress (Group C), and workers who had work-related stress but stayed on their jobs (Group D). We performed one-way ANOVA for age and total scores of IES-R and PHQ-9, General Trust Scale, and Mental Well-being Scale, followed by Bonferroni post hoc test for multiple comparisons. We employed the Chi-square test for other variables, including gender. We also tested the correlation between IES-R and PHQ-9 using Spearman's rho correlation coefficient. We conducted all analyses using SPSS for Windows, Version 22.0, and the level of significance was set at *p* < 0.05.

## Results

### Characteristics

**Fig. 1:**
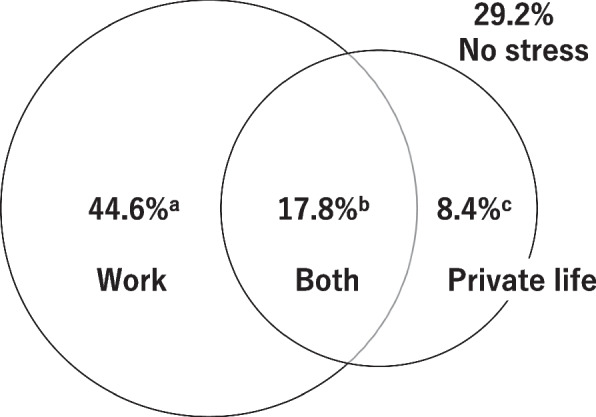
500 patients with depression or adjustment disorder who are employed and under treatment. ^**a**^ Onset with work-related stress only. ^**b**^ Onset with both work and private life stress. ^**c**^ Onset with private life stress only

The characteristics and demographic data from study 1 are presented in Table 1. As shown in Fig. 1, it turned out that within six months prior to the onset of depression or adjustment disorder, 29.2% of the participants did not experience any work-related or private life stress, while 44.6% experienced only work-related stress but no private life stress, 8.4% experienced only private life stress but no work-related stress, and 17.8% experienced both work-related and private life stress. Moreover, 30.0% of the participants answered that their stressful situations had not been improved by the time of this survey. The characteristics and demographic data from study 2 are shown in Table 2. A total of 767 workers were divided into four groups by whether or not they were diagnosed with depression, and whether or not they quit their jobs due to work-related stress: 200 workers with depression and quit their jobs (Group A), 167 workers with depression but stayed on their jobs (Group B), 200 workers without depression but quit their jobs because of work-related stress (Group C), and 200 workers with work-related stress but stayed on their jobs (Group D). 507 participants (66.1%) were male, and male participants of all groups were significantly greater than female participants (*p* < 0.001). The mean age of all groups was 48.1 years, ranging from 21 to 65 years. The one-way ANOVA was significant at the 0.05 level, F(3,763) = 5.598, *p* = 0.001. A post hoc Bonferroni test indicated that the mean age of Group A and C were significantly lower than that of Group D (*p* = 0.011, 0.001, respectively).

**Table 1:** Characteristics of the participants of Study 1

	*N* = 500	%
Age, years (SD) [Age range]	45.81 (8.96) [22–59]	-
Gender, Male	250	50.0
Marital Status, Married	273	54.6
Current employment	500	100.0
More than 2 years of disease duration	450	90.0
More than 2 years of sick leave from job	126	25.2

**Table 2:** Characteristics of the participants of Study 2

	Group A (*n* = 200)	Group B (*n* = 167)	Group C (*n* = 200)	Group D (*n* = 200)	Statistics F/χ^2^	P	Direction of difference
Age, years (SD) [Age range]	47.1 (9.82) [21–65]	48.93 (9.67) [22–65]	46.47 (9.54) [22–65]	50.12 (9.75) [25–65]	5.598^a^	0.001	A, C < D
Gender, male (%)	130 (65.0)	130 (77.8)	106 (53.0)	141 (70.5)	28.544^a^	0.001	-
Current employment (%)	133 (66.5)	151 (90.4)	155 (77.5)	177 (88.5)	44.401^a^	< 0.001	-
Mean age of resignation from the job	38.1	-	32.9 (*n* = 197)	-	0.081	< 0.001	
Mean age at onset of depression	35.3	40.0	-	-	1.448	< 0.001	
PHQ-9, mean (SD)	12.35 (7.81)	10.98 (7.33)	5.98 (6.44)	3.86 (5.01)	70.129	< 0.001	A > C, D
IES-R, mean (SD)	40.07 (23.1)	36.67 (23.4)	20.74 (21.2)	13.89 (17.4)	67.446	< 0.001	A, B > C > D
General Trust Scale (SD)	21.20 (8.24)	21.92 (8.61)	21.46 (8.00)	23.40 (8.30)	2.833	< 0.001	A > D
Mental Well-being Scale (SD)	18.58 (9.12)	19.29 (9.05)	21.70 (8.82)	23.28 (7.91)	12.084	< 0.001	A > C, D B > D
Depression with no comorbidity (%)	102 (51.0)	101 (60.5)	-	-	3.308	0.069	
Depression + PTSD (%)	22 (11.0)	9 (5.4)	-	-	1.833	0.176	
Depression + Social Anxiety Disorder (%)	27 (13.5)	15 (9.0)	-	-	0.723	0.395	
Depression + panic disorder (%)	34 (17.0)	23 (13.8)	-	-	3.705	0.054	
Depression + other mental disorder (%)	35 (17.5)	25 (15.0)	-	-	0.426	0.514	

Group A: workers who had depression and quit their jobs; Group B: workers who had depression but stayed on their jobs; Group C: workers who had no depression but quit their jobs; Group D: workers who had no depression and stayed on their jobs

^a^Chi-squared test

74.5% of Group A and 69.2% of Group B answered that their duration of treatment was more than three years. Table 3 shows the classification of stressful work-related events among all participants. The most stressful events were one-person operation for Group A (30.0%) and Group B (22.8%), workplace bullying/assault and power harassment for Group C (17.5% each), and unwanted/forced transfer for Group D (30.0%). The average age when the most stressful event occurred was 34.17 years for Group A, 38.83 years for Group B, 31.01 years for Group C, and 36.76 years for Group D.

**Table 3:** Classification of work-related stressful events

	Group A (*n* = 200)		Group B (*n* = 167)		Group C (*n* = 200)		Group D (*n* = 200)	
	N	%	N	%	N	%	N	%
Unwanted, forced transfer	43	21.5	34	20.4	30	15.0	60	30.0
One-person operation	60	30.0	38	22.8	28	14.0	49	24.5
Workplace bullying or assault	30	15.0	18	10.8	35	17.5	10	5.0
Power harassment	24	12.0	25	15.0	35	17.5	18	9.0
Serious trouble with the boss	13	6.5	19	11.4	27	13.5	13	6.5
Trouble with a colleague or subordinate	12	6.0	11	6.6	25	12.5	17	8.5
Other than the above	18	9.0	22	13.2	20	10.0	33	16.5
Total	200	100.0	167	100.0	200	100.0	200	100.0

Group A: workers who had depression and quit their jobs; Group B: workers who had depression but stayed on their jobs; Group C: workers who had no depression but quit their jobs; Group D: workers who had no depression and stayed on their jobs

### Scores of IES-R for work-related stressful events

**Fig. 2:**
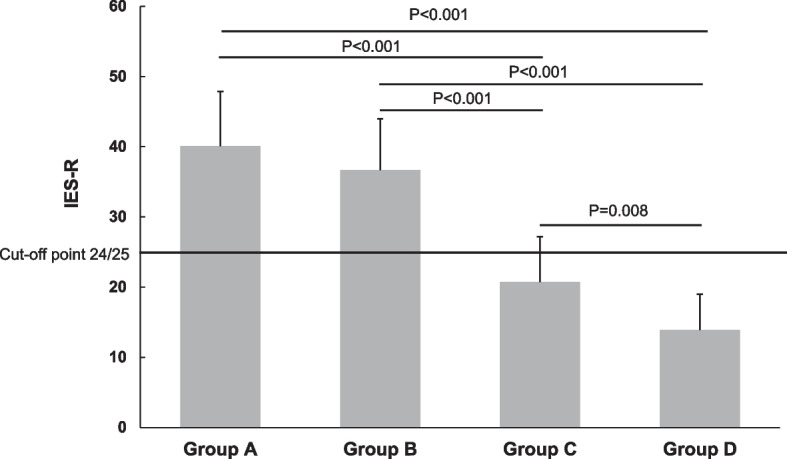
Mean score of IES-R. Group A: workers who had depression and quit their jobs; Group B: workers who had depression but stayed at their jobs; Group C: workers who did not have depression but quit their jobs due to work-related stress; Group D: workers who had work-related stress but stayed at their jobs

Figure 2 shows the IES-R scores for the most work-related stressful event for all groups. The mean scores of IES-R were 40.7 (SD = 23.1) for Group A and 36.67 (SD = 23.4) for Group B, both exceeding the cut-off point of PTSD (24/25), while the mean scores of Group C and Group D were 20.74 (SD = 21.2) and 13.89 (SD = 17.4), respectively, both below the cut-off point. The one-way ANOVA was significant at the 0.05 level, F(3,763) = 67.446, *p* < 0.001. A post hoc Bonferroni test indicated that the total scores of Groups A and B were significantly higher than those of Groups C and D (*p* < 0.001), and there was a significant difference between the two scores of Groups C and D (*P* = 0.008).

### PHQ-9 scores for the severity of depression

**Fig. 3:**
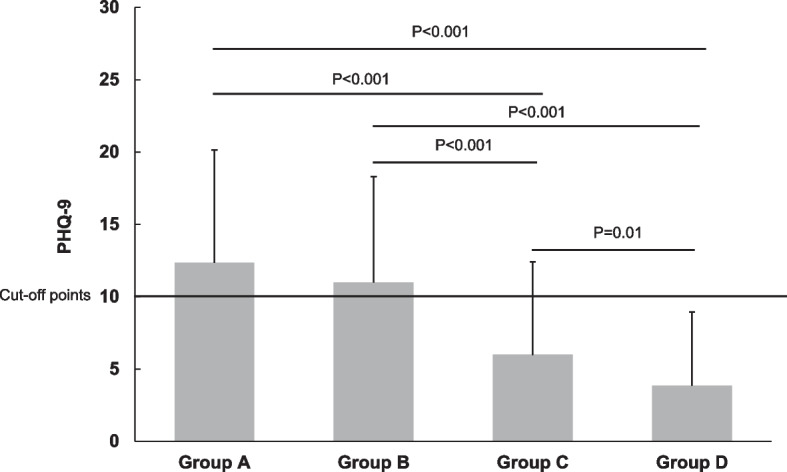
Mean score of PHQ-9. Group A: workers who had depression and quit their jobs; Group B: workers who had depression but stayed at the jobs; Group C: workers who did not have depression but quit their jobs due to work-related stress; Group D: workers who had work-related stress but stayed at their jobs

As shown in Fig. 3, the mean PHQ-9 scores were 12.35 (SD = 7.81) for Group A and 10.98 (SD = 7.33) for Group B, with both exceeding the cut-off point (10), while the mean scores of Group C and Group D were 5.98 (SD = 6.44) and 3.86 (SD = 5.01) respectively, with both below the cut-off point. The one-way ANOVA was significant at the 0.05 level, F(3,763) = 70.129, *p* < 0.001. A post hoc Bonferroni test indicated that the total scores of Groups A and B were significantly higher than those of Groups C and D (*p* < 0.001), and there was a significant difference between the scores of Group C and Group D (*p* = 0.01).

### Relationship between IES-R and PHQ-9

There was a significant positive correlation between the scores of IES-R and PHQ-9 for all four groups (*r* = 0.708).

### Scores of general trust scale

The one-way ANOVA of this scale is shown in Table 2. The mean score was 21.20 (SD = 8.24) for Group A, 21.92 (SD = 8.61) for Group B, 21.46 (SD = 8.00) for Group C, and 23.40 (SD = 8.30) for Group D. Post-hoc test revealed significant differences between Groups A and D, but there was no correlation among other groups.

### Scores of mental well-being scale

The one-way ANOVA of this scale is shown in Table 2. The mean score was 18.58 (SD = 9.12) for Group A, 19.29 (SD = 9.05) for Group B, 21.70 (SD = 8.82) for Group C, and 23.28 (SD = 7.91) for Group D. Post-hoc test revealed that the mean score of Group A was significantly higher than those of Groups C and D, and the mean score of Group B was significantly higher than that of Group D but not than those of Groups A and D. There was no significant difference between Group C and Group D.

## Discussion

In study 1, we found that more than 60% of 500 workers who were diagnosed with depression or adjustment disorder experienced work-related stress within six months prior to onset, and the onset-related stressful situation had been continuing among 30.0% of the participants. Our finding matches with previous research about relationship between depression by long work hours and sleep deprivation [19].

The results of Study 2 indicated that workers who developed depression due to work-related stressful events such as one-person operation, unwanted transfer, bullying, or power harassment had significantly higher mean scores of IES-R, which measures PTSD symptoms, than workers who did not have depression and exceeded the cut-off points. Our result corresponds with previous study which investigated correlation between victims of bullying at work and PTSD [20]. This suggests the possibility of suffering from a psychological injury caused by work-related stressful events, and it is reasonable to assume that the event itself was not physically or life threatening such as a near-death accident or a catastrophic disaster, but strongly connected with the onset of depression with symptoms of PTSD. And our finding is quite notable compared to previous findings because we additionally found out that work-related stressful events including not only bullying at work by a specific individual(s), but one-person operation or unwanted/forced transfer which doesn’t take actual “victim and perpetrator paradigm” can also be traumatic for workers and lead to development of PTSD.

On the other hand, for the result of the General Trust scale, there was no significant difference between groups of workers with depression and without depression. This means that workers with depression may not have problems of trusting others, even though previous literature suggests that major stressful life events such as bullying at work may increase symptomatology by negative evaluation of others and self [21]. Also, the Mental Well-being Scale has not been validated or standardized yet, so we need to be careful regarding any discussion, but the result implies that workers with depression had lower motivation to take care their own mental health compared to those without depression. Therefore, we need to take measures to promote mental care for depressed workers.

As far as distress and depression level of each work-related stressful events are concerned, one-person operation is perceived as the most stressful event and unwanted/forced transfer as the second ones among workers with depression, so employers need to be careful about these two events in order to prevent workers from causing psychological injury.

Taken together, these results confirm that it is desirable to measure the level of psychological injury resulting from traumatic events in the workplace because the overlooked injury may result in serious outcomes such as job resignation or development of depression. Therefore, we can conclude that it is necessary to measure not only depressive symptoms but also the level of psychological injury resulting from stressful events in the workplace. This study may provide a new perspective on what to assess when psychiatrists or occupational physicians see workers with depression and what to consider upon recovery and rehabilitation for return to work.

### Limitations

There were three limitations regarding this study. First, this was a volunteered and self-reported online survey, so it was easy to recruit a large number of participants which we considered an advantage, but sampling was not randomized, and it was possible that interpretation of the questions and answer options might differ among participants in comparison to random sampling and interview-based surveys. Second, their diagnoses were also self-reported and not based on interviews by physicians, so it was possible that the diagnosis of depression did not meet the standards of the Diagnostic and Statistical Manual of Mental Disorders, Fifth Edition (2013) [22], and there could be participants who met the criteria of the standards among those who reported not being diagnosed with depression. With regard to online-survey, there has always been an argument about data inaccuracy and deception [23], so we need to take it into consideration. Last, regarding the selection of scales, "The scores of Mental Well-being Scale" has not yet been tested for reliability and validity with a large sample set. It would be preferable to use internationally verified scales for future studies and future studies should be conducted to develop a scale to measure the level of psychological injury such as PIRI (Psychological Injury Risk Indicator) and to investigate the possibility of intervention by pharmacotherapy and evidence-based psychotherapy such as Cognitive Behavioral Therapy and how to prevent psychological injury caused in the workplace.

## Conclusion

This study showed that workers who developed depression caused by strong work-related stressful events such as one-person operation, unwanted transfer, bullying, or power harassment had significantly higher mean scores of IES-R, which measures PTSD symptoms, than those of workers who were without depression and exceeded the cut-off points. Therefore, we can conclude that it is necessary to measure not only depressive symptoms but also the level of psychological injury resulting from stressful events in the workplace.

## Data Availability

The datasets generated during and/or analyzed during the current study are available from the corresponding author on reasonable request.
